# Neutrophil extracellular trap (NET) levels in human plasma are associated with active TB

**DOI:** 10.1371/journal.pone.0182587

**Published:** 2017-08-04

**Authors:** Marcos C. Schechter, Kristina Buac, Toidi Adekambi, Stephanie Cagle, Justine Celli, Susan M. Ray, Christina C. Mehta, Balázs Rada, Jyothi Rengarajan

**Affiliations:** 1 Division of Infectious Diseases, Department of Medicine, Emory University School of Medicine, Atlanta, Georgia, United States of America; 2 Department of Infectious Diseases, College of Veterinary Medicine, University of Georgia, Athens, Georgia, United States of America; 3 Emory Vaccine Center, Department of Medicine, Emory University School of Medicine, Atlanta, Georgia, United States of America; 4 Department of Epidemiology, Rollins School of Public Health, Emory University, Atlanta, Georgia, United States of America; 5 Department of Biostatistics and Bioinformatics, Rollins School of Public Health, Emory University, Atlanta, Georgia, United States of America; Hospital for Sick Children, CANADA

## Abstract

Neutrophils are increasingly associated with tuberculosis (TB) disease. Neutrophil extracellular traps (NETs), which are released by neutrophils as a host antimicrobial defense mechanism, are also associated with tissue damage. However, a link between NET levels and TB disease has not been studied. Here we investigate plasma NETs levels in patients with active pulmonary tuberculosis using an ELISA assay that is suitable for high-throughput processing. We show that plasma NETs levels at baseline correlated with disease severity and decreased with antibiotic therapy. Our study demonstrates the biologic plausibility of measuring NETs in plasma samples from patients with TB.

## Introduction

*Mycobacterium tuberculosis* (Mtb), the etiologic agent of tuberculosis (TB), is the leading infectious cause of death worldwide [1]. Macrophages have been generally regarded as the site of Mtb infection, but neutrophils are increasingly associated with TB disease in humans [2]. Studies suggest that neutrophils mediate lung pathology and an interferon-inducible neutrophil-driven transcript signature in blood has been associated with TB clinical severity. Moreover, Mtb has been detected within neutrophils isolated from the airways of patients with active pulmonary TB patients [3–5].

Neutrophil extracellular traps (NETs) are a proposed mechanism of neutrophil antimicrobial activity that represent an important strategy to trap and kill invading microbes [6]. NETs consist of chromatin released from neutrophils that forms a scaffold with granule-derived antimicrobial peptides and enzymes to immobilize extracellular pathogens [7, 8]. Human neutrophil elastase (HNE) and myeloperoxidase (MPO) are essential for NET formation and both these enzymes are found within primary granules of resting neutrophils [8, 9]. HNE cleaves histones to unveil DNA, while MPO assists HNE to translocate to the nucleus during the process of NET formation [9]. Mtb has been shown to trigger neutrophil release of NETs *in vitro* and sputum specimens from subjects infected with Mtb contain a higher concentration of extracellular DNA compared to uninfected controls [10, 11]. While NETs were originally described as a host antimicrobial defense mechanism, NET formation has also been observed in inflammatory disorders and associated with tissue damage, suggesting an association with exacerbated inflammation and disease pathology [6, 8]. While neutrophil accumulation has been associated with TB in animal models and humans, a potential link between NET levels and human TB disease has not yet been investigated. In this study, we tested the hypothesis that NET levels correlate with TB disease.

Extracellular DNA alone is not a NET-specific measure [12]. Detection of NETs has largely relied on immunostaining and electron microscopy of DNA complexed with NET-components, which are difficult to quantitate and not conducive to high throughput use in clinical settings. To quantitate NETs, we used an ELISA assay that measures NET-specific MPO-DNA complexes in human clinical samples previously developed by the Rada group [12, 13]. Complexes of MPO-DNA derive from neutrophils releasing NETs and therefore serve as direct measures of NET formation [12, 13]. In this study, we sought to use this assay to evaluate the levels of NETs (MPO-DNA complexes), MPO and HNE in plasma from patients with active TB disease, subjects with latent TB infection (LTBI) and healthy controls (HC) with no known comorbidities (including LTBI). We found that plasma levels of NETs, MPO and HNE were significantly elevated in individuals with active TB compared to those with LTBI or HC and correlated with Mtb burden. This study demonstrates the biologic plausibility of measuring NETs in human plasma samples from TB patients [13].

## Materials and methods

### Study participants

Human immunodeficiency virus (HIV)-negative adults with respiratory culture confirmed active TB (ATB) with an inpatient admission to the Grady Memorial Hospital (Atlanta, GA, United States of America) and who were followed for ATB treatment in a local health department after hospital discharge were eligible for inclusion. When feasible, 3 plasma samples were obtained longitudinally. The first sample, referred to as “time point 0”, was collected close to the time of initiation of therapy for ATB. The second sample, referred to as “time point 1”, was designed to be collected close to the predicted time of sputum culture conversion (approximately 2 months after treatment initiation). The last sample, referred to as “time point 2”, was designed to be collected close to completion of therapy for ATB.

HIV-negative adults from Atlanta were identified as having LTBI by a positive ESAT6-CFP10-specific IFN-y ELISPOT assay as previously described [14]. HC with no known comorbidities (including LTBI) were recruited at Emory University. This study was approved by the Emory University Institutional Review Board. All participants provided written informed consent for the collection of samples and subsequent analysis.

### ELISA

Concentrations of MPO and HNE in human plasma were measured by commercial ELISA kits (Human MPO ELISA kit, R&D Systems, Minneapolis, MS, USA; Human PMN elastase ELISA kit, Abcam, Cambridge, MA, USA) following manufacturers’ instructions. Levels of MPO-DNA complexes were assessed by an MPO-DNA ELISA as described previously [12, 13]. Briefly, human plasma samples were diluted 10-fold in sterile PBS and subjected to MPO-DNA ELISA. Results are expressed as percentages of the “NET-standard” that contains pooled supernatants of PMA-stimulated human neutrophils and serves as a reference [13].

### Statistical analysis

Statistical analysis was performed using R version 3.2.3 and SAS version 9.4. Differences between non-paired samples were assessed with the Mann-Whitney *U* test. Differences between paired samples were assessed by the Wilcoxon signed-rank test. To model the longitudinal response to therapy from ATB patients, generalized estimating equation (GEE) models were used to account for the correlation of repeated measures within a patient. HNE and MPO were log-transformed for GEE models and bivariate analysis. Time on TB therapy was counted from the receipt of the first dose of TB therapy to the last dose of TB therapy, irrespective of treatment interruptions. Association between clinical parameters and markers of NET formation were assessed through correlation and ANOVA. A *P* value of less than 0.05 was considered statistically significant.

## Results

### Description of study participants

Nineteen patients with ATB were included in the study. The majority of patients with ATB were male, non-Hispanic black, and born in the United States (US) (Table 1). Patients had a high burden of disease. Thirteen (68%) patients had a high grade sputum acid fast smear (3–4+) and 13(68%) had cavitary lung disease. All patients were started on standard TB drug regimen of rifampin, isoniazid, pyrazinamide, and ethambutol under directly observed therapy (DOT) as recommended by current US guidelines [15]. Five (26%) patients had isoniazid mono-resistant TB and their drug regimens were adjusted at the discretion of the local health department physician.

**Table 1:** Baseline cohort characteristics.

Characteristic	
Male	19 (100)
Median age (IQR), years	52.4 (47–56)
US born ^a^	18 (95)
Non-Hispanic black ^b^	16 (84)
Median BMI at presentation (IQR)	20.1 (18.4–23.5)
HIV positive	0
Sputum culture positive for MTb	19 (100)
Sputum NAAT positive for MTb ^c^	16 (84)
Smear grade	
Negative	2 (11)
1–2+	4 (21)
3–4+	13 (68)
Cavitary disease	13 (68)
Largest cavity size, median, (IQR), centimeters ^d^	4.3 (2.2–4.8)
Drug susceptibility	
Isoniazid mono-resistance ^e^	5 (26)

Abbreviations: IQR, interquartile range; US, United States; BMI, body mass index; HIV, Human Immunodeficiency Virus;

MTb–*Mycobacterium tuberculosis*; NAAT, Nucleic acid amplification test

^a^ 1 subject born in India

^b^ White (n = 2), Asian (n = 1)

^c^ Data missing n = 3

^d^ Data missing n = 4

^e^ All susceptible to rifampin

**Fig 1:**
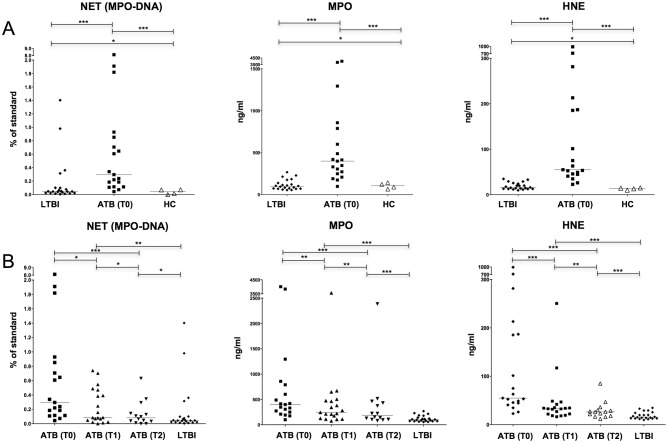
Neutrophil extracellular traps (NETs) plasma levels. Panel A: comparison of NET plasma levels among subjects with latent tuberculosis infection, healthy controls, and baseline samples of patients with active tuberculosis. Panel B: NET plasma levels during treatment for active tuberculosis and comparison with subjects with latent tuberculosis infection. Abbreviations: NET, Neutrophil Extracellular Traps; MPO, Myeloperoxidase; HNE, Human Neutrophil Elastase; LTBI, Latent Tuberculosis Infection; ATB, Active Tuberculosis; HC, Healthy Control. * P ≤ 0.05; ** P > 0.05; *** P > 0.01.

The median time between collection of plasma sample for time point 0 and initiation of TB therapy was 1 day (T0, Fig 1 and S1 Fig). Eighteen of the nineteen patients included in the study had plasma collected for time point 1 (T1, Fig 1B and S1 Fig). All patients achieved sputum culture conversion (median 15 days, IQR 1–54 days). Plasma for time point 1 was collected a median of 15 days before sputum culture conversion. Finally, fifteen of the nineteen subjects enrolled had plasma available for analysis of time point 2 (T2, Fig 1B and S1 Fig). Plasma for time point 2 was collected a median of 8.2 months after initiation of TB therapy and the median length of TB therapy was 9.7 months. DOT sheets were available for review for 16 patients with ATB. All of these 16 patients had DOT adherence above 80% for the first 3 months of TB therapy. Three patients had treatment interruption after the first 3 months of TB therapy, two for non-adherence and the other for not receiving medications while incarcerated. All patients were considered cured at the completion of TB therapy and no disease relapses have been reported to the state of Georgia health authorities.

Twenty-two individuals identified with LTBI and 4 HC were included. All individuals with LTBI were HIV-negative non-smokers with no recent history of severe respiratory disease and had normal chest x-rays. HC had no known comorbidities.

### Markers of NET formation are associated with TB disease

Plasma MPO-DNA, MPO, and HNE levels were significantly higher in patients with ATB at time point 0 compared to subjects with LTBI and HC (Fig 1A). HC and subjects with LTBI had similar plasma MPO-DNA, MPO, and HNE levels (Fig 1A).

Plasma MPO-DNA levels declined with TB therapy (Fig 1B, left). This finding was statistically significant between time points 0 and 2 and approached borderline statistical significance between time points 0 and 1 (p = 0.059). Plasma MPO-DNA levels were similar between ATB patients at time point 2 and subjects with LTBI. These data indicate that plasma NETs concentrations are associated with TB disease.

A GEE model of MPO-DNA levels was not feasible due to the 3 outliers with ATB plasma MPO-DNA levels above 100% of the assay standard at T0 (Fig 1B, left). All 3 outliers had a high grade acid-fast smear (3–4+) and cavitary disease. Two of the three outliers required intensive care unit (ICU) admission during index hospitalization. None of the other patients in the ATB cohort had an ICU admission (p = 0.02). Other markers of disease severity such as body mass index (BMI) at admission and time to sputum culture conversion were similar between these 3 outliers for plasma MPO-DNA levels and the other 16 patients with ATB.

Plasma MPO and HNE levels had a statistically significant linear trend of decline over the 3 time points (Fig 1B, middle and right). Plasma MPO and HNE levels remained elevated at time point 2 compared to subjects with LTBI and HC. This difference was statistically significant for all comparisons, except for plasma MPO levels between time point 2 and HC (p = 0.06). Plasma MPO and HNE trends were not associated with white blood cell (WBC) and/or neutrophil trends both alone or in combination (data not shown). This association could not be tested for MPO-DNA as GEE modeling was not feasible. There was no association between age, BMI at TB diagnosis, smear grade, presence of cavity, or cavity size and markers of NET formation at baseline by bivariate analysis (data not shown).

## Discussion

Our results show that active TB in human patients is associated with higher systemic concentrations of the following neutrophil markers: NETs (MPO-DNA), MPO, and HNE indicating that robust neutrophil activation accompanies TB disease and can also be tracked in the plasma. These data suggest that NET formation could be a major contributor to the reported rapid neutrophil cell death during acute TB [2]. NET release during Mtb infection may promote immunopathology, for example by promoting the development of caseous granulomatous lesions in the lung [4]. These observations strengthen accumulating evidence that neutrophils play a major role in TB pathogenesis [2]. Previously MPO and HNE have been detected in sputa and BAL samples of TB patients but our data showing their easy detection in the blood suggests that they may have utility as potential biomarkers of active TB. Moreover, our data showing decreased NET levels in plasma following chemotherapy, mirror antibiotic-mediated clearance of Mtb.

Neutrophils have recently attracted increased attention from the TB scientific community, however their role *in vi*vo in human TB remains poorly understood. Neutrophils were shown to release NETs *in vitro* when exposed to Mtb [10]. Our data showing measurable levels of NETs in the plasma of ATB patients indicate that NET formation occurs *in vivo* in patients with TB disease. MPO-DNA complexes present in plasma only detect NETs, but do not indicate whether NETs have been generated in neutrophils in an NADPH oxidase-dependent or–independent manner. *In vitro* NET release stimulated by Mtb or BCG coincides with robust superoxide production in neutrophils [10, 16, 17]. The NADPH oxidase inhibitor diphenylene iodonium inhibited Mtb-induced NET formation similarly to that stimulated by the oxidase activator phorbol-myrystate-acetate [18]. These data suggest that NETs present in plasma of patients with active TB were generated in neutrophils by an oxidative mechanism. NADPH oxidase-independent, calcium-dependent NET release is mediated by protein arginine deiminase 4 (PAD4) and produces citrullinated histones in the process [19, 20]. Whether citrullinated histones are elevated or unchanged in patients with active TB, remains an open question.

NETs are only one of the several activation markers of neutrophils. Neutrophils were shown to release metalloproteases and cytokines in response to Mtb [11, 21]. Whether NET formation is the mechanism for their release, remains to be studied. Neutrophils can release MPO and HNE also by degranulation. The fact that levels of both, total MPO and MPO-DNA complexes behave similarly in our study and neutrophils do not release MPO *in vitro* by degranulation [16], suggests that most plasma MPO is released by NET formation in active TB.

## Conclusion

We report enhanced levels of NETs in human plasma during ATB compared to LTBI and decreased NET levels in ATB following anti-TB therapy. Inhibiting NET release in conjunction with antibiotic therapy could be clinically beneficial and future mechanistic studies are required to further reveal the contribution of NETs to disease pathogenesis.

## Supporting information

S1 Fig Study scheme. (TIF)

## References

[1] WHO. Global tuberculosis report 2016. WHO Library Cataloguing-in-Publication Data. 2016;WHO/HTM/TB/2016.13.

[2] Dallenga T, Schaible UE. Neutrophils in tuberculosis—first line of defence or booster of disease and targets for host-directed therapy? Pathog Dis. 2016;74(3). doi: 10.1093/femspd/ftw012 . 26903072 10.1093/femspd/ftw012

[3] Eum SY, Kong JH, Hong MS, Lee YJ, Kim JH, Hwang SH, et al Neutrophils are the predominant infected phagocytic cells in the airways of patients with active pulmonary TB. Chest. 2010;137(1):122–8. doi: 10.1378/chest.09-0903 ; 19749004 10.1378/chest.09-0903 PMC2803122

[4] Gopal R, Monin L, Torres D, Slight S, Mehra S, McKenna KC, et al S100A8/A9 proteins mediate neutrophilic inflammation and lung pathology during tuberculosis. Am J Respir Crit Care Med. 2013;188(9):1137–46. doi: 10.1164/rccm.201304-0803OC ; 24047412 10.1164/rccm.201304-0803OC PMC3863739

[5] Berry MP, Graham CM, McNab FW, Xu Z, Bloch SA, Oni T, et al An interferon-inducible neutrophil-driven blood transcriptional signature in human tuberculosis. Nature. 2010;466(7309):973–7. doi: 10.1038/nature09247 ; 20725040 10.1038/nature09247 PMC3492754

[6] Sorensen OE, Borregaard N. Neutrophil extracellular traps—the dark side of neutrophils. J Clin Invest. 2016;126(5):1612–20. doi: 10.1172/JCI84538 ; 27135878 10.1172/JCI84538 PMC4855925

[7] Brinkmann V, Reichard U, Goosmann C, Fauler B, Uhlemann Y, Weiss DS, et al Neutrophil extracellular traps kill bacteria. Science. 2004;303(5663):1532–5. doi: 10.1126/science.1092385 . 15001782 10.1126/science.1092385

[8] Bardoel BW, Kenny EF, Sollberger G, Zychlinsky A. The balancing act of neutrophils. Cell Host Microbe. 2014;15(5):526–36. doi: 10.1016/j.chom.2014.04.011 . 24832448 10.1016/j.chom.2014.04.011

[9] Papayannopoulos V, Metzler KD, Hakkim A, Zychlinsky A. Neutrophil elastase and myeloperoxidase regulate the formation of neutrophil extracellular traps. J Cell Biol. 2010;191(3):677–91. doi: 10.1083/jcb.201006052 ; 20974816 10.1083/jcb.201006052 PMC3003309

[10] Ramos-Kichik V, Mondragon-Flores R, Mondragon-Castelan M, Gonzalez-Pozos S, Muniz-Hernandez S, Rojas-Espinosa O, et al Neutrophil extracellular traps are induced by Mycobacterium tuberculosis. Tuberculosis (Edinb). 2009;89(1):29–37. doi: 10.1016/j.tube.2008.09.009 . 19056316 10.1016/j.tube.2008.09.009

[11] Ong CW, Elkington PT, Brilha S, Ugarte-Gil C, Tome-Esteban MT, Tezera LB, et al Neutrophil-Derived MMP-8 Drives AMPK-Dependent Matrix Destruction in Human Pulmonary Tuberculosis. PLoS pathogens. 2015;11(5):e1004917 doi: 10.1371/journal.ppat.1004917 ; 25996154 10.1371/journal.ppat.1004917 PMC4440706

[12] Yoo DG, Floyd M, Winn M, Moskowitz SM, Rada B. NET formation induced by Pseudomonas aeruginosa cystic fibrosis isolates measured as release of myeloperoxidase-DNA and neutrophil elastase-DNA complexes. Immunol Lett. 2014;160(2):186–94. doi: 10.1016/j.imlet.2014.03.003 . 24670966 10.1016/j.imlet.2014.03.003

[13] Sil P, Yoo DG, Floyd M, Gingerich A, Rada B. High Throughput Measurement of Extracellular DNA Release and Quantitative NET Formation in Human Neutrophils In Vitro. J Vis Exp. 2016;(112). doi: 10.3791/52779 . 27404503 10.3791/52779 PMC4993224

[14] Adekambi T, Ibegbu CC, Kalokhe AS, Yu T, Ray SM, Rengarajan J. Distinct effector memory CD4+ T cell signatures in latent Mycobacterium tuberculosis infection, BCG vaccination and clinically resolved tuberculosis. PLoS One. 2012;7(4):e36046 doi: 10.1371/journal.pone.0036046 ; 22545156 10.1371/journal.pone.0036046 PMC3335801

[15] Nahid P, Dorman SE, Alipanah N, Barry PM, Brozek JL, Cattamanchi A, et al Official American Thoracic Society/Centers for Disease Control and Prevention/Infectious Diseases Society of America Clinical Practice Guidelines: Treatment of Drug-Susceptible Tuberculosis. Clin Infect Dis. 2016 doi: 10.1093/cid/ciw376 . 27516382 10.1093/cid/ciw376 PMC6590850

[16] Arcos J, Diangelo LE, Scordo JM, Sasindran SJ, Moliva JI, Turner J, et al Lung Mucosa Lining Fluid Modification of Mycobacterium tuberculosis to Reprogram Human Neutrophil Killing Mechanisms. The Journal of infectious diseases. 2015;212(6):948–58. doi: 10.1093/infdis/jiv146 ; 25748325 10.1093/infdis/jiv146 PMC4548464

[17] Tenland E, Hakansson G, Alaridah N, Lutay N, Ronnholm A, Hallgren O, et al Innate Immune Responses after Airway Epithelial Stimulation with Mycobacterium bovis Bacille-Calmette Guerin. PloS one. 2016;11(10):e0164431 doi: 10.1371/journal.pone.0164431 ; 27723804 10.1371/journal.pone.0164431 PMC5056730

[18] Braian C, Hogea V, Stendahl O. Mycobacterium tuberculosis- induced neutrophil extracellular traps activate human macrophages. Journal of innate immunity. 2013;5(6):591–602. doi: 10.1159/000348676 . 23635526 10.1159/000348676 PMC6741595

[19] Li P, Li M, Lindberg MR, Kennett MJ, Xiong N, Wang Y. PAD4 is essential for antibacterial innate immunity mediated by neutrophil extracellular traps. J Exp Med. 2010;207(9):1853–62. doi: 10.1084/jem.20100239 ; 20733033 10.1084/jem.20100239 PMC2931169

[20] Wang Y, Li M, Stadler S, Correll S, Li P, Wang D, et al Histone hypercitrullination mediates chromatin decondensation and neutrophil extracellular trap formation. J Cell Biol. 2009;184(2):205–13. doi: 10.1083/jcb.200806072 ; 19153223 10.1083/jcb.200806072 PMC2654299

[21] Cai S, Batra S, Langohr I, Iwakura Y, Jeyaseelan S. IFN-gamma induction by neutrophil-derived IL-17A homodimer augments pulmonary antibacterial defense. Mucosal Immunol. 2016;9(3):718–29. doi: 10.1038/mi.2015.95 ; 26349661 10.1038/mi.2015.95 PMC4785101

