# Supplementary figures and images for: Neutrophil extracellular trap (NET) levels in human plasma are associated with active TB
Source: PLoS One. 2017 Aug 4;12(8):e0182587. doi: 10.1371/journal.pone.0182587 (PMC5544211; doi:10.1371/journal.pone.0182587)

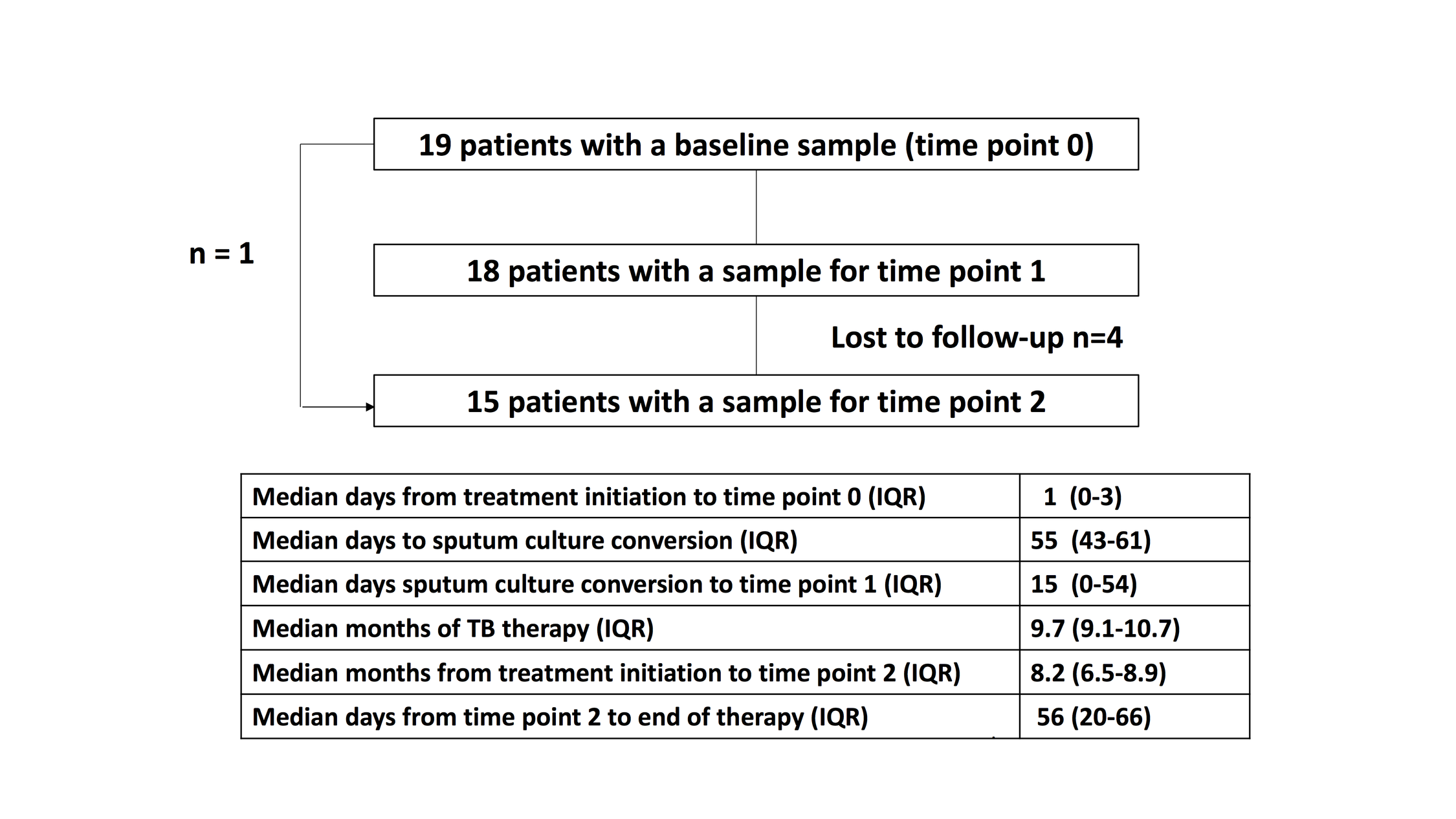

Supplement: S1 Fig — (TIF) [file pone.0182587.s001.tif]
